# Increased Incidence of Dysmenorrhea in Women Exposed to Higher Concentrations of NO, NO_2_, NO_x_, CO, and PM_2.5_: A Nationwide Population-Based Study

**DOI:** 10.3389/fpubh.2021.682341

**Published:** 2021-06-17

**Authors:** Shih-Yi Lin, Yu-Cih Yang, Cheng-Chieh Lin, Cherry Yin-Yi Chang, Wu-Huei Hsu, I-Kuan Wang, Chia-Der Lin, Chung-Y. Hsu, Chia-Hung Kao

**Affiliations:** ^1^Graduate Institute of Biomedical Sciences and School of Medicine, College of Medicine, China Medical University, Taichung, Taiwan; ^2^Division of Nephrology and Kidney Institute, China Medical University Hospital, Taichung, Taiwan; ^3^Management Office for Health Data, China Medical University Hospital, Taichung, Taiwan; ^4^College of Medicine, China Medical University, Taichung, Taiwan; ^5^Department of Family Medicine, China Medical University Hospital, Taichung, Taiwan; ^6^Department of Gynecology, China Medical University Hospital, Taichung, Taiwan; ^7^Department of Chest Medicine, China Medical University Hospital, Taichung, Taiwan; ^8^Department Teaching, China Medical University Hospital, Taichung, Taiwan; ^9^Department Otolaryngology, China Medical University Hospital, Taichung, Taiwan; ^10^Department of Nuclear Medicine and PET Center, China Medical University Hospital, Taichung, Taiwan; ^11^Department of Bioinformatics and Medical Engineering, Asia University, Taichung, Taiwan; ^12^Center of Augmented Intelligence in Healthcare, China Medical University Hospital, Taichung, Taiwan

**Keywords:** air pollution, dysmenorrhea, Taiwan air quality monitoring database, national health insurance research database, NOX, CO, PM_2.5_

## Abstract

**Background:** Air pollution is speculated to affect the reproductive health of women. However, a longitudinal association between exposure to air pollution and dysmenorrhea has not been identified, which this study aimed to examine this point.

**Methods:** Two nationwide databases, namely the Taiwan Air Quality Monitoring database and the Taiwan National Health Research Institutes database were linked. Women with a history of dysmenorrhea (International Classification of Disease, Ninth Revision, Clinical Modification code 625.3) before 2000 were excluded. All participants were followed from January 1, 2000 until the diagnosis of dysmenorrhea, withdrawal from National Health Insurance, or December 31, 2013. Furthermore, air pollutants were categorized into quartiles with three cut-off points (25th, 50th, and 75th percentiles). The Cox regression model was used to calculate the hazard ratios of dysmenorrhea.

**Results:** This study enrolled 296,078 women. The mean concentrations of yearly air pollutants were 28.2 (±12.6) ppb for nitric oxides (NO_x_), 8.91 (±7.93) ppb for nitric oxide (NO), 19.3 (±5.49) ppb for nitrogen dioxide (NO_2_), 0.54 (±0.18) ppm for carbon monoxide (CO), and 31.8 (±6.80) μg/m^3^ for PM_2.5_. In total, 12,514 individuals developed dysmenorrhea during the 12-year follow-up. Relative to women exposed to Q1 concentrations of NO_x_, women exposed to Q4 concentrations exhibited a significantly higher dysmenorrhea risk [adjusted hazard ratio (aHR)= 27.9, 95% confidence interval (CI) = 21.6–31.3]; similarly higher risk was found for exposure to NO (aHR = 16.7, 95% CI = 15.4–18.4) and NO_2_ (aHR = 33.1, 95% CI = 30.9–37.4). For CO, the relative dysmenorrhea risk in women with Q4 level exposure was 28.7 (95% CI = 25.4–33.6). For PM_2.5_, women at the Q4 exposure level were 27.6 times (95% CI = 23.1–29.1) more likely to develop dysmenorrhea than those at the Q1 exposure level.

**Conclusion:** Our results showed that women would have higher dysmenorrhea incidences while exposure to high concentrations of NO, NO_2_, NO_x_, CO, and PM_2.5_.

## Introduction

Dysmenorrhea, characterized by painful cramps of the uterus during menstruation, affects 45–95% of menstruating women. Dysmenorrhea is the leading morbidity among gynecological disorders and the leading cause of pelvic pain (1), thus representing the greatest burden for menstruating women (2, 3). Moreover, dysmenorrhea is related to negative performance in school and social and sports activities as well as short-term school absenteeism among adolescents (4). Furthermore, dysmenorrhea affects the economy insofar as it impairs work productivity (5), and almost 600 million working hours are lost yearly due to dysmenorrhea (6). It is classified as either primary dysmenorrhea, which does not involve a related organic disease, or secondary dysmenorrhea, which is related to attributable causes such as endometriosis (7). Although the actual causes of primary dysmenorrhea are unclear, the most well-known pathogenesis is prostaglandin overproduction in the uterus, particularly overproduction of PGF_2a_ and PGF_2_ (8). Therefore, the mechanism of primary dysmenorrhea involves arachidonic acid (AA) and inflammation (9), and thus, the effective treatment for primary dysmenorrhea is non-steroidal anti-inflammatory drugs and cyclooxygenase (COX) inhibitors (10, 11).

Mahalingaiah et al. (12) and Carré et al. (13) observed that all size fractions of particulate matter (PM) exposure and traffic-related air pollution are associated with incidence of infertility and adverse reproductive health (14). Merklinger-Gruchala et al. (15) and Mahalingaiah et al. (16) showed that the mean concentrations of the air pollutants particles 10 microns and below(PM_10_), Sulfur dioxide(SO_2_), Nitrogen Oxides (NO_x_), and Carbon monoxide (CO) were associated with shortening of the luteal phase, an irregular menstrual cycle, menstrual disorders and menstrual irregularity, respectively. The above evidence suggested that although infertility and irregular menstrual cycle had many different anatomic or hormonal causes, they would be affected by air pollutants. Therefore, air pollutants might have effect on menstruation. However, to the best of our knowledge, no epidemiological study has investigated the association between primary dysmenorrhea and air pollutants.

Therefore, we used nationwide medical records and nationwide air pollution monitoring databases to conduct a retrospective cohort study to determine whether exposure to air pollutants is associated with high dysmenorrhea risk.

## Methods

### Data Source

To clarify dysmenorrhea risk in participants exposed to air pollutants, we used the Longitudinal Health Insurance Database 2000 (LHID 2000) from the Taiwan National Health Insurance Research Database (NHIRD), which includes ~99% of the population in Taiwan. The LHID 2000 consists of 1 million patients who were randomly selected from NHIRD in 2000. LHID 2000 contains comprehensive deidentified health care information regarding patient demographics, outpatient visits, inpatient care, prescription drugs, and medical procedures from 1996 to 2013. The National Health Research Institutes confirmed that no significant differences were evident in the distribution of age, sex, or health care costs between the population in the LHID 2000 and that in the NHIRD. This study used the International Classification of Disease, Ninth Revision, Clinical Modification (ICD-9-CM) to categorize disease diagnoses based on inpatient data.

Taiwan Environmental Protection Administration (EPA) have set up 76 air quality observation stations nationwide for the Air Quality Observation and Forecast Network at the end of 2006 (17) (Supplementary Figure 1) According to different pollution characteristics, weather conditions, and observational purposes of EPA, the air quality observation stations were distributed reasonably in cities and countries and divided into seven main island regions and three outlying islands regions (18) (Supplementary Figure 1). Geographic information system (GIS) (ArcGIS version 10; ESRI, Redlands, CA, USA) was used to identify and manage the locations of the monitoring stations and air pollution sources. The monitoring data were integrated into yearly point data and interpolated to pollutant surfaces using inverse distance weighting method (IDW) (19).

We obtained air pollution data [nitric oxides (NO_x_), nitric oxide (NO), nitrogen dioxide (NO_2_), carbon monoxide (CO), and PM_2.5_. from the Taiwan Air Quality Monitoring Database (TAQMD). The TAQMD provides daily air pollution data from community-based monitoring sites that are available in real-time on the organizations' website. We downloaded data for the pollutants NO_x_, NO, NO_2_, CO, and PM_2.5_ and calculated their annual mean levels from January 2000 to December 2012. To define average exposure duration of women to these pollutants is really an issue. If exposure duration to air pollutants was defined too shorter (e.g., <1 year or only several months), the results that air pollutants is associated with risks of dysmenorrheal would not be convincing. In the other aspect, if duration was defined too long (e.g., longer than 10 or 5 years), the unknown and uncontrolled bias (i.e., body weight change, take hormone, change of residence, or pregnancy, etc) would be too much to make the results convincing. To avoid and minimize such bias, we defined duration of average exposure levels of women to these pollutants as 2 years. Then, we computed the average exposure levels of women to these pollutants for 2 years before the diagnosis of dysmenorrhea or end of the study period for each individual.

### Sample Participants

**Figure 1:**
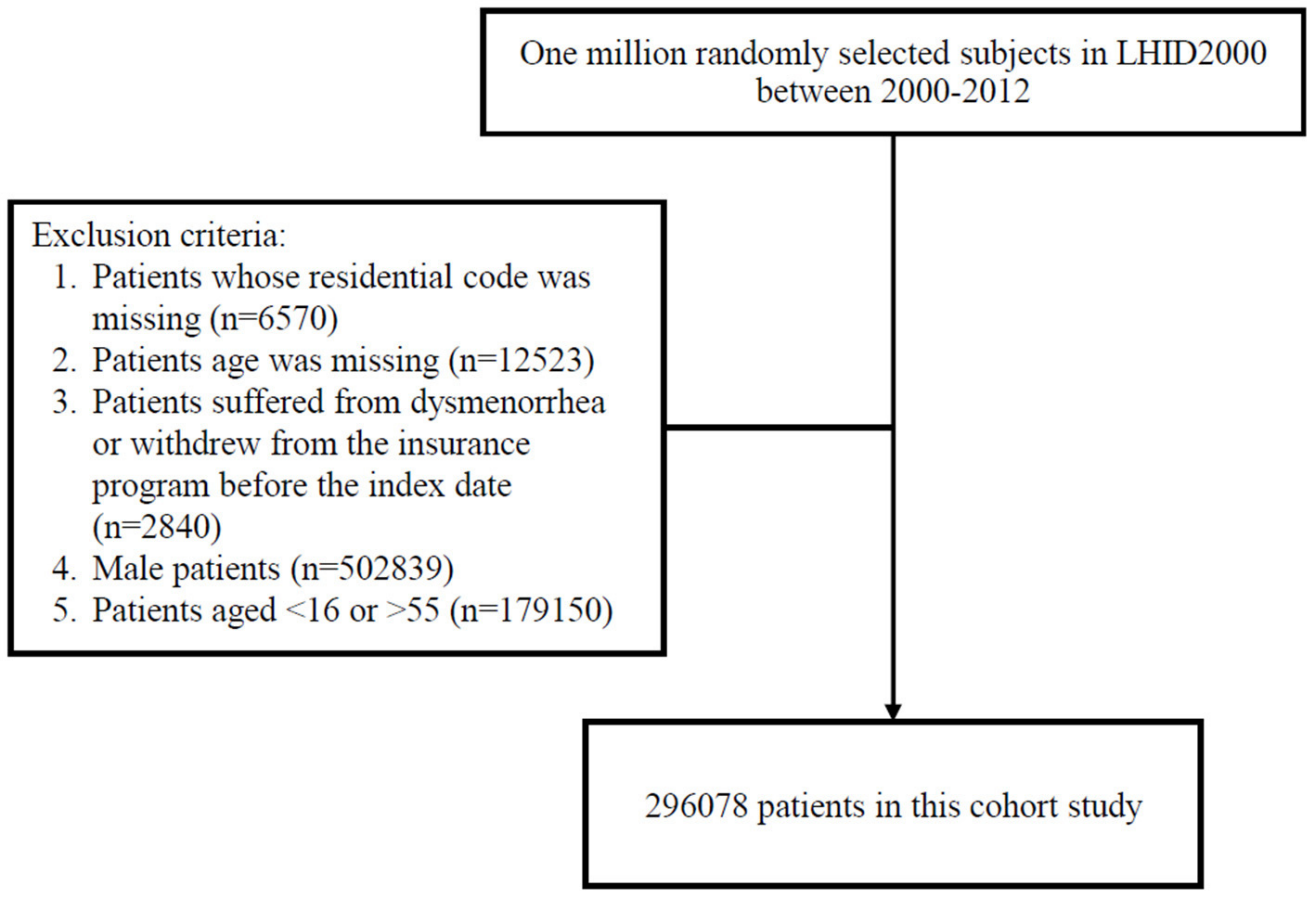
Flow chart of this study.

Women residing in areas with air quality monitoring stations were the focus of this study, which representative of exposure. However, women with a history of dysmenorrhea (ICD-9-CM code 625.3) (http://www.icd9data.com/2012/Volume1/580-629/617-629/625/625.3.htm) before 2000 were excluded. All participants were 16–55 years-old and were followed from January 1, 2000 until dysmenorrhea diagnosis (ICD-9-CM code 625.3), withdrawal from National Health Insurance, or December 31, 2013 (Figure 1). The confounding factors were age, sex, urbanization level of residence, monthly income, occupational class (20, 21), and comorbidities such as hypertension (ICD-9-CM 401–405), diabetes mellitus (ICD-9-CM 250), hyperlipidemia (ICD-9-CM 272), heart disease (ICD-9-CM 410–414), Chronic obstructive pulmonary disease (COPD) (ICD-9-CM 490–496), chronic kidney disease (ICD-9-CM 580–589), stroke (ICD-9-CM 430–438), depression (ICD-9-CM 296.2, 296.3, 296.82, 300.4, 309.0, 309.1, 309.28, and 311), chronic liver disease, and cirrhosis (ICD-9-CM 070.6, 070.9, 570, 571, 573.3, 573.4, 573.8, 573.9, and V42.7). Causes of secondary dysmenorrhea were also considered as covariates included endometriosis (ICD-9-CM 617), pelvic inflammatory disease (ICD-9-CM 614 and 615), ovarian cancer (ICD-9-CM 183.0), inflammatory bowel disease (ICD-9-CM 555 and 556), and intramural fibroids or intracavitary fibroids (ICD-9-CM 218). The National Health Research Institutes has stratified all city districts and townships in Taiwan into seven urbanization levels on the basis of population density (people/km^2^); the proportion of residents with higher education, and who work in agriculture; and the number of physicians per 100,000 people in each area. Level 1 represents areas with a high population density and socioeconomic status, and level 7 represents areas with the lowest socioeconomic status. Because few people live in the rural areas classified as levels 4–7, our study grouped these areas into level 4. Monthly income was classified into 3 groups: < NT$15,000, NT$15,000–29,999, and >NT$30,000. Occupational class was divided into three classes: white collar, blue collar, and other class. White-collar occupations involve working in an office and doing work that requires mental rather than physical effort. Blue-collar workers, such as farmers or fishermen, do work that requires strength or physical skill. The other class includes unemployed people, soldiers, and religious people.

### Outcome and Exposure Measurement

The study endpoint was the diagnosis of dysmenorrhea (ICD-9-CM code 625.3). We integrated daily concentrations of air pollutants corresponding to residential zip codes to calculate 2-year average exposure before the diagnosis of dysmenorrhea and the year of diagnosis of dysmenorrhea from 2000 to 2012.To estimate the effect of air pollutants on dysmenorrhea, we investigated the daily concentrations of NO_x_, NO, NO_2_, CO, and PM_2.5_ by using the inverse distance weighting (IDW) method. IDW is a common and simple spatial interpolation method. It predicts values of unknown points on the basis of the similarity of two objects in terms of distance (22). Its principle is to use distance measurement to perform the weighted analysis of the interpolation. When the unknown point is estimated to be closer to the known point, the weighted value of the unknown point will be higher. Hence, when the distribution of the known measuring point is balanced, the value estimated using the IDW method will be more accurate (Supplementary Table 1; Supplementary Figure 1) (23, 24). In this study, the generalized additive model (GAM) was used to quantitatively evaluate the acute effects of air pollution including NOx, NO, NO2, PM_2.5_, and CO concentrations and interquartile range on the incidence of dysmenorrhea in Taiwan. We found that the plots of the generalized additive model for interquartile range more linear than the plots of generalized additive model for air pollution concentrations. In this study, the air pollutant exposure levels were scaled to the interquartile range (IQR). Furthermore, Air pollutants were also categorized into quartiles with three cut-off point (25th, 50th and 75th percentiles) for NOx (Quartile 1, <19.1 ppb; Quartile 2, 19.1–24.7 ppb; Quartile 3, 24.8–34.5 ppb; Quartile 4, >34.6 ppb), NO (Quartile 1, <4.0 ppb; Quartile 2, 4.0–5.52 ppb; Quartile 3, 5.53-11.5 ppb; Quartile 4, >11.6 ppb), NO_2_ (Quartile 1, <15.2 ppb; Quartile 2, 15.2–18.8 ppb; Quartile 3, 18.9–22.8 ppb; Quartile 4, >22.9 ppb), PM_2.5_ (Quartile 1, <27.1 μg/m^3^; Quartile 2, 27.1–32.0 μg/m^3^; Quartile 3, 32.1–35.3 μg/m^3^; Quartile 4, > 35.4 μg/m^3^), and CO (Quartile 1, <0.42 ppm; Quartile 2, 0.42–0.49 ppm; Quartile 3, 0.50–0.59 ppm; Quartile 4, > 0.60 ppm).

### Statistical Analysis

We conducted a Cox proportional hazards regression for the multivariable analysis of dysmenorrhea diagnosis through increase in IQR (μg/m^3^ or ppb) of long-term exposure to NO_x_, NO, NO_2_, CO, and PM_2.5_ from 2000 to dysmenorrhea development or the end of the study period (25).

Dysmenorrhea incidence (per 10,000 person-years) was calculated at each level of air pollutant concentration. The relative risk of dysmenorrhea in participants who were exposed to Q2–Q4 levels of air pollutant concentrations relative to those exposed to the Q1 level of air pollutant concentrations was estimated using the Cox proportional hazards regression model. The potential confounders, namely age, monthly income, urbanization level, occupational class, and comorbidities, were defined as determinants of dysmenorrhea and associated with air pollution concentrations and further incorporated into models. Furthermore, we used the Cox regression model, which is stratified by age, urbanization level, monthly income, and occupational class, for analyzing dysmenorrhea risk between various yearly average concentrations of air pollutants. This study uses Generalized additive model (GAM) to analyze the impact of air pollutant on dysmenorrhea (26, 27). GAM is a non-parametric extension of Generalized linear model (GLM) (28). It not only retains the basic framework of GLM, but also has many assumptions as other linear models (such as normal assumption or variance homogeneity), and its ability to handle non-linear models is better than other models (29). It is powerful and suitable for dealing with overly complex and non-linear relationships among many variables. In this study, air pollution data are usually non-linear, which limits the application of GLM, so it can be solved through GAM (30). The aim of this study was to use GAM to evaluate the relation between air pollution and dysmenorrheal by using data from the NHIR database and TAQM database (296,078 participants of whom 12,514 died during 11.7 years follow-up). Using GAM, we found that strong positive association between air pollution and dysmenorrhea, the association also appeared in low concentrations of air pollution (Supplementary Figure 2).

## Results

There were total 296,078 participants in this study, and there were 12,514 participants had diagnosis of dysmenorrhea. Table 1 demonstrates the baseline characteristics and air pollutant exposure of the study cohort. Among the 296,078 female subjects, insured persons in 30–44 years of age (41.7%), living in north area (48.0%), living in urbanization level 1 (33.4%), monthly income <15,000–29,999 (47.3%), and white collar class worker (57.9%) were dominant. The mean age of the study subjects was 33.7 (±10.4) years old. The mean of the follow up time was 11.7 (±1.37) years. The mean of yearly air pollutants concentration was 28.2 (±12.6) ppb for NOx, 8.91 (±7.93) ppb for NO, 19.3 (±5.49) ppb for NO_2_, 31.8 (±6.80) μg/m^3^ for PM_2.5_, and 0.54 (±0.18) ppm for CO. There were 12,514 subjects developed dysmenorrhea after mean 11.7-years follow-up. Among 296,078 patients, there were 44,384 patients (14.9%) used oral contraceptives.

**Table 1:** Baseline demographics and exposure of air pollutants in Taiwan.

***N* = 296,078**	***n***	**%**
**Age, years**		
<30	119,509	40.4
30–44	123,563	41.7
≥45	53,006	17.9
Mean, (SD)	33.7 (10.4)	
**Area**		
North	142,247	48
Central	58,917	19.9
Southern	73,839	24.9
Eastern	21,075	7.12
**Urbanization level**		
1 (highest)	98,907	33.4
2	90,526	30.6
3	51,666	14.4
4 (low)	54,979	18.6
**Monthly income**		
<15,000	118,317	39.9
15,000–29,999	140,161	47.3
≥3,0000	37,600	12.7
**Occupational class**		
White color class	171,502	57.9
Blue color class	102,163	34.5
Other	22,413	7.57
**Comorbidity**		
Hypertension	50,567	17.1
Diabetes mellitus	44,452	15
Hyperlipdemia	49,321	16.7
Heart disease	21,433	7.24
COPD	57,139	19.3
Chronic kidney disease	11,630	3.93
Stroke	11,757	3.97
Chronic liver disease and cirrhosis	44,848	15.1
Endometriosis	236	0.08
Depression	24,766	8.36
Pelvic inflammatory disease	80,175	27
Ovary cancer	838	0.28
Inflammatory bowel disease	7,598	2.57
Intramural fibroids or intracavity fibroids	37,467	12.6
Pregnancy before index date	91,843	31
**NO**_**x**_ **level (daily average, ppb)**		
Mean, SD^†^	28.2 (12.6)	
Min	4.18	
Lower quartile	19.1	
Median	24.8	
Upper quartile	34.6	
Max	127	
**NO level (daily average, ppb)**		
Mean, SD^†^	8.91 (7.93)	
Min	0.46	
Lower quartile	4	
Median	5.53	
Upper quartile	11.6	
Max	85.3	
**NO**_**2**_ **level (daily average, ppb)**		
Mean, SD^†^	19.3 (5.49)	
Min	2.71	
Lower quartile	15.2	
Median	18.9	
Upper quartile	22.9	
Max	41.6	
**PM**_**2.5**_ **level (daily average**, **μg/m**^**3**^**)**		
Mean, SD^†^	31.8 (6.80)	
Min	12.1	
Lower quartile	27.1	
Median	32.1	
Upper quartile	35.4	
Max	70.6	
**CO level (daily average, ppm)**		
Mean, SD^†^	0.54 (0.18)	
Min	0.12	
Lower quartile	0.42	
Median	0.5	
Upper quartile	0.6	
Max	2.29	
**Outcome**		
Dysmenorrhea	12,514	4.23
Follow-up time, years (mean, SD)	11.7 (1.37)	

*NOx, nitrogen oxides; NO, nitrogen monoxide; NO2, nitrogen dioxide; CO, Nitric oxide; PM_2.5_, particle matter with diameter less 2.5 μm; SD, standard deviation*.

*The urbanization level was categorized by the population density of the residential area into 4 levels, with level 1 as the most urbanized and level 4 as the last urbanized*.

*COPD, Chronic obstructive pulmonary disease*.

† *standard deviation*.

The distribution of urbanization level among different quartile of air pollutant levels was displayed in Supplementary Table 2. Participants exposed to the Q4 level air pollutant including NOx, NO, NO_2_, PM_2.5_, and CO mostly lived in the level 1 urbanization area.

Table 2 shows the incidence rate of dysmenorrhea among levels of all air pollutant concentrations. After controlling for potential confounding factors, Q4 air pollutant level exposure had higher risk of dysmenorrhea significantly compared with Q1 level of air pollutants. For NOx, relative to Q1 concentrations, the Q4 (adjusted = 27.9, 95%CI = 21.6–31.3) concentrations were had a significant higher risk of dysmenorrhea. Relative to Q1 NO concentrations, the Q4 (adjusted = 16.7, 95% CI = 15.4–18.4) also had a significant higher risk of dysmenorrhea. Relative to Q1 NO_2_ concentrations, the Q4 (adjusted = 33.1, 95% CI = 30.9–37.4) also had a significant higher risk of dysmenorrhea. The relative risks of dysmenorrhea in Q4 level exposure were 27.6 (95% CI = 23.1–29.1) for PM_2.5_. People under Q4 CO level exposure had 28.7 (95% CI = 25.4–33.6) fold increased risk to develop dysmenorrhea than those under Q1 level.

**Table 2:** Difference in dysmenorrhea incidences and associated HRs in participant exposed to various daily average concentration of air pollutants.

**Pollutant levels**	***N***	**Dysmenorrhea**	**PY**	**IR**	**Crude HR**	**Adjusted HR**
		**event**			**(95% CI)**	**(95% CI)**
**NOx (daily average)**						
Q1	78,548	1,386	966,921	1.43	1 (reference)	1 (reference)
Q2	71,830	2,379	850,647	2.8	1.89 (1.77–2.02)^***^	2.45 (2.23–2.89)^***^
Q3	73,111	3,714	855,434	4.34	2.93 (2.76–3.12)^***^	7.53 (7.13–8.67)^***^
Q4	72,589	5,035	838,164	6.01	4.06 (3.83–4.31)^**^	27.9 (21.6–31.3)^***^
**NO (daily average)**						
Q1	79,248	1,629	943,949	1.73	1 (reference)	1 (reference)
Q2	71,936	1,892	854,517	2.21	1.28 (1.20–1.37)^***^	1.58 (1.45–2.01)^***^
Q3	64,187	4,285	744,069	5.76	3.34 (3.15–3.53)^***^	7.13 (6.54–8.81)^***^
Q4	80,707	4,708	938,631	5.02	2.90 (2.74–3.07)^***^	16.7 (15.4–18.4)^***^
**NO**_**2**_ **(daily average)**						
Q1	78,568	1,589	936,256	1.7	1 (reference)	1 (reference)
Q2	68,096	2,197	806,817	2.72	1.60 (1.50–1.71)^***^	1.57 (1.46–2.17)^***^
Q3	77,089	2,753	910,337	3.02	1.78 (1.67–1.89)^***^	5.33 (4.19–6.58)^***^
Q4	72,325	5,975	827,756	7.22	4.25 (4.02–4.50)^***^	33.1 (30.9–37.4)^***^
**PM**_**2.5**_ **(daily average)**						
Q1	78,978	2,228	932,916	2.39	1 (reference)	1 (reference)
Q2	70,207	3,018	828,248	3.64	1.52 (1.44–1.61)^***^	1.19 (1.06–1.45)^***^
Q3	74,411	1,575	886,486	1.78	0.74 (0.49–0.79)^***^	1.17 (1.01–1.64)^***^
Q4	72,482	5,693	833,516	6.83	2.83 (2.72–3.00)^***^	27.6 (23.1–29.1)^***^
**CO (daily average)**						
Q1	75,223	1,232	898,940	1.37	1 (reference)	1 (reference)
Q2	69,275	1,570	825,979	1.9	1.38 (1.28–1.49)^***^	1.78 (1.46–2.13)^***^
Q3	75,707	2,528	895,579	2.82	2.06 (1.92–2.20)^***^	7.31 (6.58–8.29)^***^
Q4	75,873	7,184	860,668	8.35	6.09 (5.74–6.47)^***^	28.7 (25.4–33.6)^***^

*Q1, first quartile; Q2, second quartile; Q3, third quartile; Q4, fourth quartile; PY, person-years; IR, incidence rate, per 1,000 person-years; HR, hazard ratio; CI, confidence interval*.

*HR adjusted for age, monthly income, urbanization level, occupational class, and comorbidities*.

** *P < 0.01*;

*** *P < 0.001*.

Table 3 showed the result of stratified Cox regression analysis by considering subject exposed to Q1 level of air pollutants as reference group. Relative to Q1 concentrations of NOx, NO, NO_2_, PM_2.5_, and CO, the participants who exposed in Q4 level of air pollutants had a significant higher risk of dysmenorrhea in each age group, monthly income, occupational class, and urbanization level 1, 2, and 3. Participants who aged 30–44 exposed to Q4 quartile of NOx and NO had 33.7 (95% CI = 28.4–36.5) fold increased risk and 21.0 (95% CI = 14.7–23.9) fold increased risk to develop dysmenorrhea, respectively, than those under Q1 level. Participants who aged <30 exposed to Q4 quartile of PM_2.5_ and CO had 29.1 (95% CI = 26.7–35.5) fold increased risk and 27.7 (95% CI = 23.6–33.5) fold increased risk, respectively, to develop dysmenorrhea than those under Q1 level. Participants lived in urbanization level 4 (low) exposing to Q 4 level of NOx, NO, and CO had highest risk of dysmenorrhea with 31.3 (95% CI = 19.3–36.7) fold, 63.2 (95% CI = 48.5–89.9) fold, 51.7 (95% CI = 33.3–60.1) fold, and 48.4 (95% CI = 26.7–54.8) accordingly, compared with exposing to Q1 level. Participants with monthly income 15000-29999 exposing to Q4 level of PM_2.5_ had 37.0 (95% CI = 27.8–53.2) fold risk of dysmenorrhea compared with exposing to Q1 level of PM_2.5_. Participants with monthly income <15,000 exposing to Q4 level of NOx had 38.1 (95% CI = 29.1–41.7) fold risk of dysmenorrhea compared with exposing to Q1 level of PM_2.5_.

**Table 3:** Incidence rate and hazard ratio of dysmenorrhea between various daily average concentrations of NOx, NO, NO_2_, PM_2.5_, CO stratified by gender, age, and comorbidities.

	**Adjusted HR (95%CI)**					
**Air pollutants**		**NOx**	**NO**	**NO_**2**_**	**PM_**2.5**_**	**CO**
**IQR**	**Quartile1 (lowest)**	**Quartile 4 (highest)**	**Quartile 4 (highest)**	**Quartile 4 (highest)**	**Quartile 4 (highest)**	**Quartile 4 (highest)**
**Age, years**						
<30	1 (reference)	31.0 (29.4–33.5)^***^	17.1 (13.2–20.6)^***^	35.4 (27.8–43.2)^***^	29.1 (26.7–35.5)^***^	27.7 (23.6–33.5)^***^
30–44	1 (reference)	33.7 (28.4–36.5)^***^	21.0 (14.7–23.9)^***^	36.5 (29.1–53.2)^***^	23.4 (15.4–29.8)^***^	21.5 (17.7–36.5)^***^
≥45	1 (reference)	9.58 (7.65–13.2)^***^	10.7 (6.52–13.7)^***^	9.43 (7.37–10.9)^***^	4.13 (3.13–8.15)^***^	7.71 (4.53–10.3)^***^
**Urbanization level**						
1 (highest)	1 (reference)	11.4 (9.43–13.5)^***^	3.96 (2.16–8.17)^***^	21.4 (16.9–29.4)^***^	31.5 (29.7–44.1)^***^	10.1 (4.39–12.6)^***^
2	1 (reference)	25.3 (20.1–28.9)^***^	15.4 (11.8–17.4)^***^	43.1 (37.1–49.5)^***^	25.7 (21.5–45.9)^***^	20.9 (13.7–31.8)^***^
3	1 (reference)	25.4 (17.3–39.5)^***^	20.9 (11.5–23.9)^***^	13.7 (10.8–20.1)^***^	32.4 (29.8–45.7)^***^	35.7 (32.2–54.3)^***^
4 (low)	1 (reference)	31.3 (19.3–36.7)^***^	63.2 (48.5–89.9)^***^	51.7 (33.3–60.1)^***^	6.93 (4.13–8.17)^***^	48.4 (26.7–54.8)^***^
**Monthly income**						
<15,000	1 (reference)	38.1 (29.1–41.7)^***^	16.9 (12.1–21.8)^***^	36.5 (30.1–47.3)^***^	28.5 (23.1–36.9)^***^	30.1 (26.4–31.7)^***^
15,000–29,999	1 (reference)	29.7 (26.3–38.4)^***^	15.7 (13.6–22.2)^***^	37.0 (27.8–53.2)^***^	21.7 (18.6–23.6)^***^	20.4 (13.6–29.7)^***^
≥30,000	1 (reference)	24.1 (13.5–29.6)^***^	17.4 (11.1–29.8)^***^	27.8 (20.0–43.1)^***^	17.8 (13.5–27.8)^***^	17.3 (16.5–22.3)^***^
**Occupational class**						
White color class	1 (reference)	29.1 (24.5–36.7)^***^	14.5 (11.3–21.6)^***^	34.4 (27.1–45.9)^***^	46.5 (31.7–49.6)^***^	21.2 (19.8–25.9)^***^
Blue color class	1 (reference)	30.1 (19.4–35.2)^***^	16.7 (12.1–20.9)^***^	33.5 (30.8–39.9)^***^	5.43 (3.13–7.68)^***^	31.7 (27.7–36.9)^***^
Other	1 (reference)	34.5 (29.2–84.5)^***^	48.6 (43.5–69.8)^***^	64.5 (49.4–87.8)^***^	24.5 (19.8–32.6)^***^	40.1 (29.8–70.4)^***^

*Q1, first quartile; Q2, second quartile; Q3, third quartile; Q4, fourth quartile; PY, person-years; IR, incidence rate, per 1,000 person-years; HR, hazard ratio; CI, confidence interval*.

*HR adjusted for age, monthly income, urbanization level, occupational class, and comorbidities*.

*** *P < 0.001*.

Participants whose occupational class as others exposed to Q4 quartile of NOx, NO, and NO_2_ had increased risk of dysmenorrheal up to 34.5 (95% CI = 29.2–84.5), 48.6 (95% CI = 43.5–69.8), 64.5 (95% CI = 49.4–87.8) fold accordingly compared with those under Q1 level. Supplementary Figures 3–7 provides illustrations of these results.

Table 4 showed associated HRs in participants exposed to every increment of one unit of yearly average concentrations of air pollutants. For every increment of 1 unit yearly average of NOX, the adjusted HR of dysmenorrhea would be increased to 1.51. For every increment of 1 unit yearly average of NO2, the adjusted HR of dysmenorrhea would be increased to 1.72. For every increment of 1 unit yearly average of NO, the adjusted HR of dysmenorrhea would be increased to 1.53. For every increment of 1 unit yearly average of CO, the adjusted HR of dysmenorrhea would be increased to 1.009. For every increment of 1 unit yearly average of PM_2.5_, the adjusted HR of dysmenorrhea would be increased to 1.81.

**Table 4:** Associated HRs in participants exposed to every increment of one unit of yearly average concentrations of air pollutants.

**Pollutant levels, unit**	**cHR**	**95%CI**	**aHR**	**95%CI**
**Dysmenorrhea**				
NOx, per1 ppb	1.62	(1.56–1.69)^***^	1.51	(1.45–1.58)^***^
NO_2_, per1 ppb	1.67	(1.64–1.70)^***^	1.72	(1.69–1.75)^***^
NO, per1 ppb	1.51	(1.49–1.53)^**^	1.53	(1.51–1.56)^***^
CO, per1 ppm	1.005	(1.005–1.006)^*^	1.009	(1.008–1.011)^**^
PM_2.5_, per1 μg/m^3^	1.85	(1.85–1.86)^*^	1.81	(1.80–1.81)^*^

*PY, Person-years; cHR, crude hazard ratio; aHR, adjusted hazard ratio of a multivariate analysis, after adjustment for age, monthly income, urbanization level, occupation class, and comorbidities; CI, confidence interval*.

* *P < 0.05*;

** *P < 0.01*;

*** *P < 0.001*.

Table 5 showed the synergy effects of PM_2.3_ and NOx, NO, NO_2_, and CO on the risks of dysmenorrhea. The cut value was median value of air pollutant which exposure concentration being less than median value was defined as low and being more than median value was defined as high. Women exposed to low PM_2.5_ and high NOx had 1.30-fold adjusted risk of dysmenorrhea (95% CI = 1.21–1.39), compared with those exposure to low PM_2.5_ and low NOx. Women exposed to low PM_2.5_ and high NO had 2.64-fold adjusted risk of dysmenorrhea (95% CI = 2.46–2.84), compared with those exposure to low PM_2.5_ and low NO. Women exposed to low PM_2.5_ and high NO_2_ had 3.75-fold adjusted risk of dysmenorrhea (95% CI = 3.84–4.05), compared with those exposure to low PM_2.5_ and low NO_2_. Women exposed to low PM_2.5_ and high CO had 5.03-fold adjusted risk of dysmenorrhea (95% CI = 4.67–5.42), compared with those exposure to low PM_2.5_ and low CO. Women exposed to high PM_2.5_ and high concentrations of NOx, NO, NO_2_, and CO had highest risk of dysmenorrhea in each category, which 10.8-fold adjusted risk of dysmenorrhea (95% CI = 10.0–11.7) for NO_X_, 6.46-fold adjusted risk of dysmenorrhea (95% CI = 6.01–6.94) for NO, 9.77-fold adjusted risk of dysmenorrhea (95% CI = 9.06–10.5) for NO_2_, and 13.0-fold adjusted risk of dysmenorrhea (95% CI = 12.1–13.9) for CO.

**Table 5:** Cox proportional hazard regression analysis for the interactive effects of PM_2.5_ with NOx, NO, NO_2_, and CO on the risk of dysmenorrhea association.

**Variables of air pollutions**		**N**	**Dysmenorrhea event**	**Crude HR (95%CI)**	**Adjusted HR (95%CI)**	**P for interaction**
**PM**_**2.5**_	**NOx**					0.13
Low	Low	63,263	1,416	1 (Reference)	1 (Reference)	
Low	High	84,145	3,612	1.94 (1.82–2.06)^***^	4.05 (3.75–4.38)^***^	
High	Low	114,345	3,718	1.46 (1.37–1.55)^***^	1.30 (1.21–1.39)^***^	
High	High	34,325	3,768	5.19 (4.88–5.51)^***^	10.8 (10.0–11.7)	
**PM**_**2.5**_	**NO**					0.56
Low	Low	70,312	1,664	1 (Reference)	1 (Reference)	
Low	High	77,096	3,364	1.87 (1.76–1.98)^***^	2.64 (2.46–2.84)^***^	
High	Low	122,375	5,020	1.75 (1.65–1.85)^***^	1.25 (1.17–1.34)^***^	
High	High	26,295	2,466	4.14 (3.89–4.41)^***^	6.46 (6.01–6.94)^***^	
**PM**_**2.5**_	**NO2**					0.27
Low	Low	60,952	1,307	1 (Reference)	1 (Reference)	
Low	High	86,456	3,721	2.03 (1.91–2.17)^***^	3.75 (3.48–4.05)^***^	
High	Low	94,266	2,737	1.36 (1.27–1.45)^***^	1.43 (1.33–1.53)^***^	
High	High	54,404	4,749	4.24 (3.99–4.51)^***^	9.77 (9.06–10.5)	
**PM**_**2.5**_	**CO**					0.68
Low	Low	70,684	1,371	1 (Reference)	1 (Reference)	
Low	High	76,724	3,657	2.50 (2.35–2.66)^***^	5.03 (4.67–5.42)^***^	
High	Low	99,304	2,410	1.25 (1.17–1.33)^***^	1.15 (1.07–1.23)^***^	
High	High	49,366	5,076	5.60 (5.28–5.94)^***^	13.0 (12.1–13.9)^***^	

*cHR, crude hazard ratio; aHR, adjusted hazard ratio of a multivariate analysis, after adjustment for age, monthly income, urbanization level, occupation class, and comorbidities; Low, concentrations of air pollutant less than median value; High, concentrations of air pollutant more than median value*.

*** *P < 0.001*.

## Discussion

Our results demonstrated that women exposed to relatively high concentrations of NO, NO_2_, NO_x_, CO, and PM_2.5_ had relatively high incidences of dysmenorrhea. This was the first cohort study to identify the association between dysmenorrhea and exposure to certain air pollutants.

Since no previous studies have reported association between air pollution and dysmenorrhea, there was no available direct information to be cited scientifically for our findings. However, in recent years, air pollution has been reported to be associated with many diseases, which resulting from direct injures to lung as well as provoking systematic inflammation (31, 32). Pope et al. reported that exposure to PM_2.5_ was associated with endothelial injury and systemic inflammation, which involves increased thrombotic propensity, loss of apoptosis of endothelial cells, and changes in cytokines (33). Chen et al. showed that exposure to fine-particle air pollutants was associated with increased urinary metabolites, namely AA, which is indicative of the inflammatory property of air pollution (34). Therefore, systematic inflammation and associated increasing oxidative stress would be plausible pathway for association between air pollution and dysmenorrhea.

We proposed several possible mechanisms accounting for our research findings: (1) increased prostaglandin synthesis (35, 36); (2) increased oxidative stress as well as myeloperoxidase activation (37–42); (3)irregular menstrual cycle (15, 16); and (4) increased emotional stress in those patients exposed to higher concentrations of air pollutions (43).

First, increased levels of 8-epi-PGF2α have been detected in exhaled breath and urine after exposure to air pollution PM_2.5_ (44–48). Furthermore, Schneider et al. and Samet et al. found that air pollution could activate macrophages, leading to the production of prostaglandin E2 and induce induce prostaglandin H synthase 2 (35, 49). Yan et al. found that NO_2_ inhalation could promote COX-2 elevation-mediated prostaglandin E2 production (36). Sang et al. showed that sulfur dioxide (SO_2_) could induce cyclooxygenases-2-derived prostaglandin E2 production and its downstream signaling pathway in neurons (50). Therefore, exposing to air pollution NOx, NO2, and PM_2.5_ could cause increasing levels of prostaglandin E2 production within body. Since increasing levels of prostaglandin E2 had been related with dysmenorrhea (51, 52), we supposed that it would be one possible explanation pathway for our findings.

Second, myeloperoxidase activation plays a role in the association between air pollution and increased incidence of dysmenorrhea. Kaplan et al. summarized that myeloperoxidase as well as oxidative stress and calcium ion levels are involved in the pathogenesis of primary dysmenorrhea (53). Also, oxidative stress and increased serum levels of the cytokines malondialdehyde and interleukin-6 are associated with dysmenorrhea (54). Constantin showed that SO_2_ and NO_2_ induce oxidative burst and myeloperoxidase activation (37). Moreover, several studies have shown an association between air pollution and oxidative stress in humans (38–42). Szmidt et al. have systematic reviewed that oxidative stress was associated with development of dysmenorrhea (55). Results of Szmidt et al. indicated an elevated level of oxidative stress, especially of lipid peroxidation existed among dysmenorrhea women (55). Yang et al. have found that exposure to PM_2.5_ will cause dysregulation of lipid metabolism and oxidation (56). Therefore, women exposure to NO_2_, NO_X_, as well as PM_2.5_ might lead to increasing oxidative stress, therefore had higher risks of dysmenorrhea.

Third, an irregular menstrual cycle is also associated with dysmenorrhea in women exposed to high concentrations of the air pollutants SO_2_ and NO_x_. Merklinger-Gruchala et al. (15) and Mahalingaiah et al. (16) found that the concentrations of the air pollutants were associated with shortening of the luteal phase, an irregular menstrual cycle, and menstrual irregularity. An irregular menstrual cycle is also a risk factor for dysmenorrhea (57), and therefore, air pollution might lead to dysmenorrhea through irregular menstruation. Additionally, Beck revealed that CO intoxication is related to dysmenorrhea, menorrhagia, and amenorrhea in women (58). Although we have adjusted for irregular menses, irregular menses might also be speculated as one plausible pathway for air pollution related dysmenorrhea since not all patients with irregular menses would seek for medical therapy.

Finally, emotional stress resulting from exposure to air pollution may contribute to increased incidence of dysmenorrhea. Power et al. reported that exposure to fine particulate air pollution was associated with anxiety (43). Further, extensive long-term exposure to air pollution may increase the odds of depression, antidepressant use, and mental health problems in children (59, 60). Since mental stress has been reported to be a risk factor for dysmenorrhea (61–64), therefore exposure to air pollution might increase mental stress and then dysmenorrhea occurrs.

Another novel interesting finding is that our results showed synergy effects of PM_2.5_ and NO, NO2, NOx, CO on the risks of dysmenorrheal. The risks of dysmenorrhea appeared to be highest in women exposed to high concentrations of NO, NO2, NOx, CO and high PM_2.5_. We supposed that it would be that high concentrations of PM_2.5_ and high concentrations of acid gases could provoke the highest oxidative stress as well as systemic inflammation. Further, the risk of dysmenorrhea would be generally higher if women exposed to high concentrations of NO, NO2, NOx, or CO, despite women exposed to low or high concentrations of PM_2.5_. This observational finding warrants attention since it implied that NO, NO2, NOx, or CO would have more harms for risks of dysmenorrheal. We suppose that high concentrations of NO, NO2, NOx, or CO would also have effects on epigenetic modifications (65). Further studies are required to explore the underlying mechanisms.

This study has several limitations. First, the NHIRD does not provide information regarding risk factors for dysmenorrhea, including family history of dysmenorrhea, an early age of menarche (younger than 12 years), nulliparity, heavy menses or irregular menses, smoking, obesity, dietary habits, life stress, use of oral contraceptives, and social network support (20, 66). Second, although National Health Insurance covers up to 99% of the population, patients with dysmenorrhea might take over-the-counter medications instead of visiting a doctor. Therefore, the incidences of dysmenorrhea would be underdiagnosed in all quartiles of air pollutants. Therefore, it should be cautious in interpreting results of this study. Third, although we have considered pelvic inflammatory disease, cervical stenosis and polyps, functional ovarian cysts, malignant ovarian tumors, and inflammatory bowel disease, we did not consider adenomyosis, inflammation and scarring of uterus, intracavity or intramural fibroids, functional ovarian cysts, and intrauterine devices, which are also associated with dysmenorrhea (67). Forth, although there is considerable difference between primary and secondary dysmenorrhea, information about hormone measurement, regularity of menses, distribution of body fat, and signs of hirsutism were unavailable in NHIRD. Fifth, although we have used zip codes to define individual exposure to air pollutants, misclassification of exposure measurement should occur in this study since each participant was free mobile. Sixth, considerations for analyzing designs inclusive 1-year exposure average window, IDW methods, as well as GAM for categorical variables should be announced here. Since this is a retrospective study, it would be many uncontrolled variables generating during this exposure window such as those causes of secondary dysmenorrhea. If exposure window was too long, it would be more hardly to convince the association between air pollution and dysmenorrhea even if results are of statistical significance. Thus, although a 1-year exposure average window seems too short for dysmenorrhea investigation, we decided 1-year as exposure average window. The association between air pollution and primary dysmenorrhea would be more convincing on the basis on current design of 1-year exposure average window. The advantage of using IDW method is that IDW is assumed substantially that the rate of correlations and similarities between neighbors is proportional to the distance between them that can be defined as a distance reverse function of every point from neighboring points. However, on an area of 36,193 square kilometers of Taiwan, the density of 78 air quality monitoring stations is not very high. Thus, another limitation of this study would be limited numbers of air pollution monitoring stations. The principal advantage of GAM is its ability to model highly complex non-linear relationships when the number of potential predictors is large. The disadvantage of GAM is that like other non-parametric methods, GAM has a high propensity for overfitting. Finally, this retrospective study was based on analysis of existing database, therefore the level of internal bias of this study would be more compared with those prospective randomized control trial studies. However, our study demonstrated real-world observational results.

In conclusion, our study showed that exposure to high concentrations of the air pollutants SO_2_, NO_x_, NO, and NO_2_ was associated with increased incidences of dysmenorrhea. Whether personal protection against air pollution could help lessen the risk of dysmenorrhea will require further investigations.

## Data Availability Statement

The datasets presented in this article are not readily available because The dataset used in this study is held by the Taiwan Ministry of Health and Welfare (MOHW). The Ministry of Health and Welfare must approve our application to access this data. Any researcher interested in accessing this dataset can submit an application form to the Ministry of Health and Welfare requesting access. Please contact the staff of MOHW (Email: stcarolwu@mohw.gov.tw) for further assistance. Taiwan Ministry of Health and Welfare Address: No. 488, Sec. 6, Zhongxiao E. Rd., Nangang Dist., Taipei City 115, Taiwan (R.O.C.). Phone: +886-2-8590-6848. All relevant data are within the paper. Requests to access the datasets should be directed to Please contact the staff of MOHW (Email: stcarolwu@mohw.gov.tw) for further assistance.

## Ethics Statement

This study was approved to fulfill the condition for exemption by the Institutional Review Board (IRB) of China Medical University (CMUH104-REC2-115-CR4). The IRB also specifically waived the consent requirement. Written informed consent for participation was not required for this study in accordance with the national legislation and the institutional requirements.

## Author Contributions

S-YL and C-HK: conception/design and collection and/or assembly of data. C-HK: provision of study materials. S-YL, W-HH, C-CL, I-KW, and C-HK: data analysis and interpretation. S-YL, Y-CY, C-CL, CC, W-HH, I-KW, C-DL, C-YH, and C-HK: manuscript writing and final approval of manuscript. All authors contributed to the article and approved the submitted version.

## Conflict of Interest

The authors declare that the research was conducted in the absence of any commercial or financial relationships that could be construed as a potential conflict of interest.

## Supplementary Material

Supplementary Figure 1 Maps of monitoring stations of air pollutants in Taiwan.

Supplementary Figure 2 The plots of the generalized additive models showed great deviation from a linear relationship.

Supplementary Figure 3 Kaplan-Meier curve about NOx exposure and dysmenorrhea risk.

Supplementary Figure 4 Kaplan-Meier curve about NO exposure and dysmenorrhea risk.

Supplementary Figure 5 Kaplan-Meier curve about NO_2_ exposure and dysmenorrhea risk.

Supplementary Figure 6 Kaplan-Meier curve about CO exposure and dysmenorrhea risk.

Supplementary Figure 7 Kaplan-Meier curve about PM_2.5_ exposure and dysmenorrhea risk.

Supplementary Table 1 Description correlation matrix for air pollutants. ^***^correlation is significant at the <0.0001 level (two tails).

Supplementary Table 2 Baseline urbanization level among quartiles of daily average concentration of air pollutants in Taiwan. *P*-value using chi-square for the comparisons between urbanization level among quartiles of daily average concentration of air pollutants. The urbanization level was categorized by the population density of the residential area into 4 levels, with level 1 as the most urbanized and level 4 as the last urbanized. The daily average air pollutant concentrations were categorized based on quartiles for each air pollutants.
