# Supplementary figures and images for: Increased Incidence of Dysmenorrhea in Women Exposed to Higher Concentrations of NO, NO2, NOx, CO, and PM2.5: A Nationwide Population-Based Study
Source: Front Public Health. 2021 Jun 17;9:682341. doi: 10.3389/fpubh.2021.682341 (PMC8247898; doi:10.3389/fpubh.2021.682341)

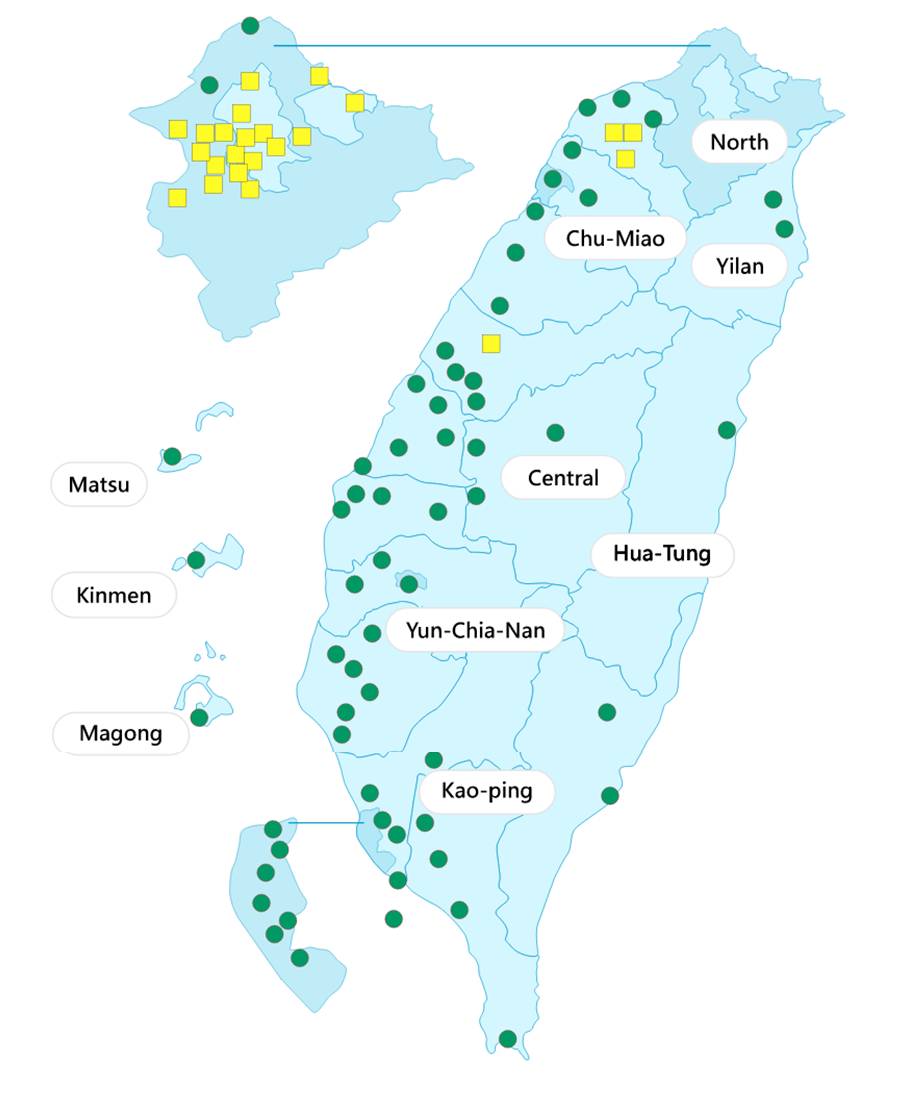

Supplement: Supplementary Figure 1 — Maps of monitoring stations of air pollutants in Taiwan. [file Image_1.JPEG]

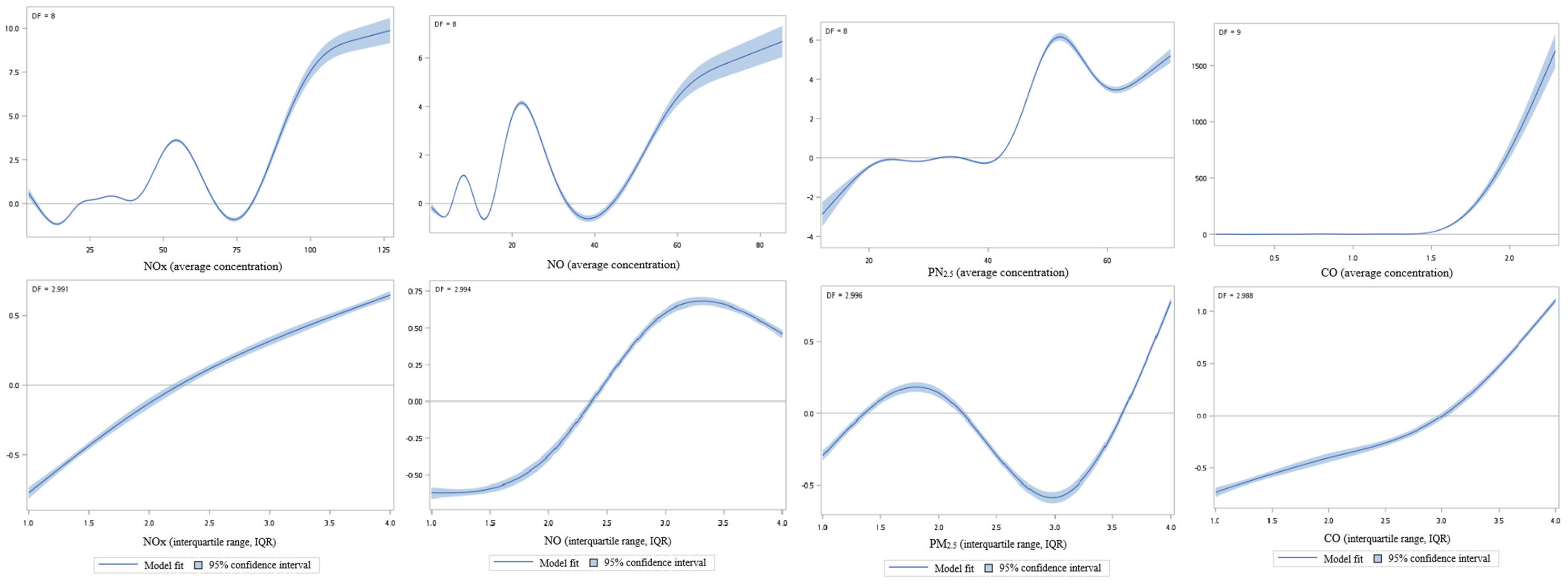

Supplement: Supplementary Figure 2 — The plots of the generalized additive models showed great deviation from a linear relationship. [file Image_2.TIF]

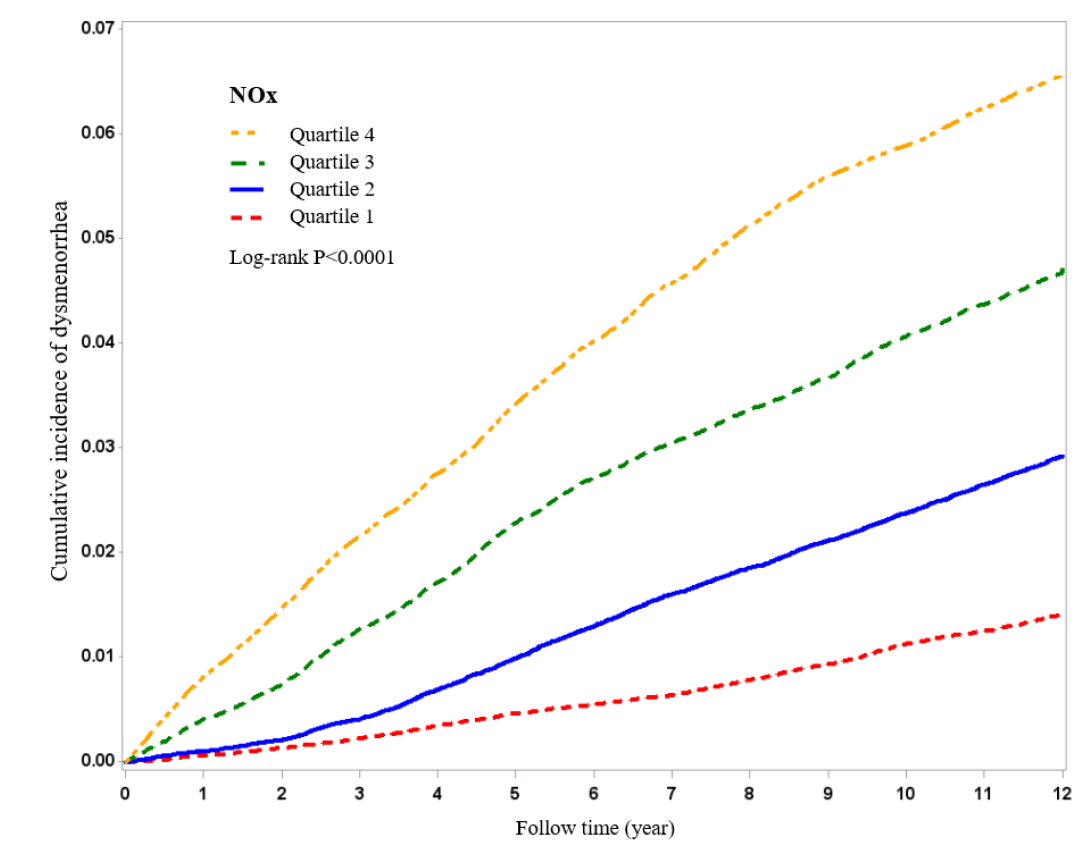

Supplement: Supplementary Figure 3 — Kaplan-Meier curve about NOx exposure and dysmenorrhea risk. [file Image_3.TIF]

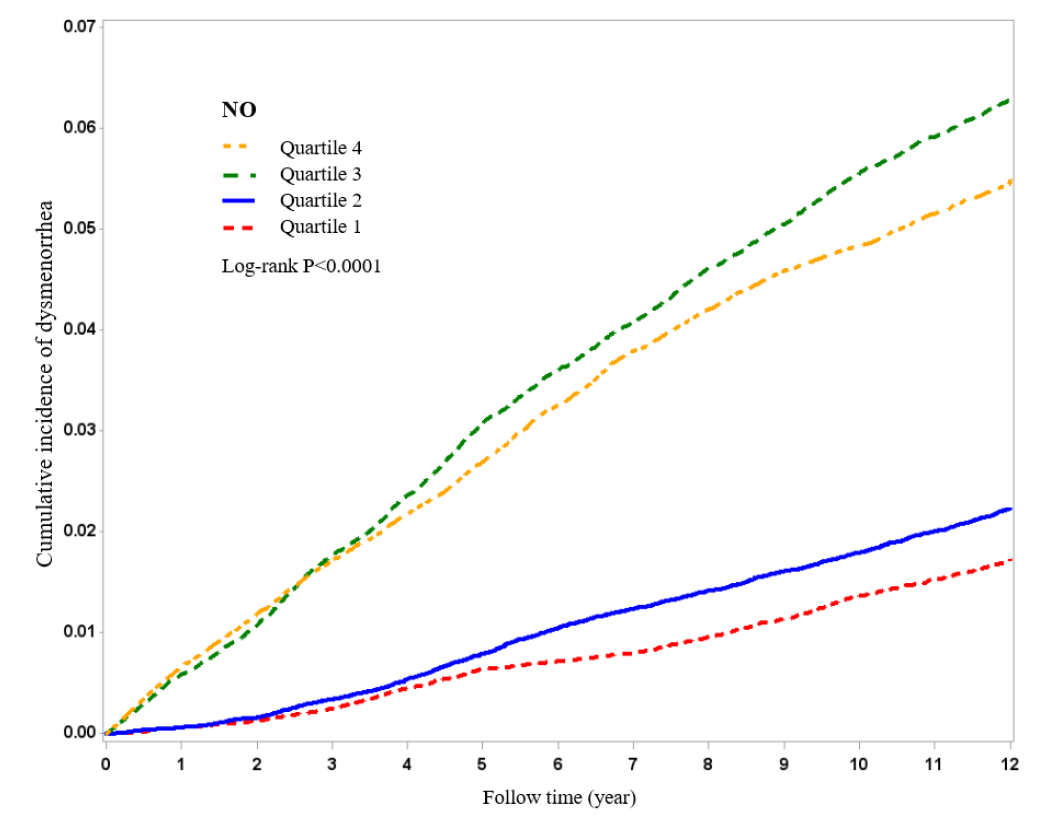

Supplement: Supplementary Figure 4 — Kaplan-Meier curve about NO exposure and dysmenorrhea risk. [file Image_4.TIF]

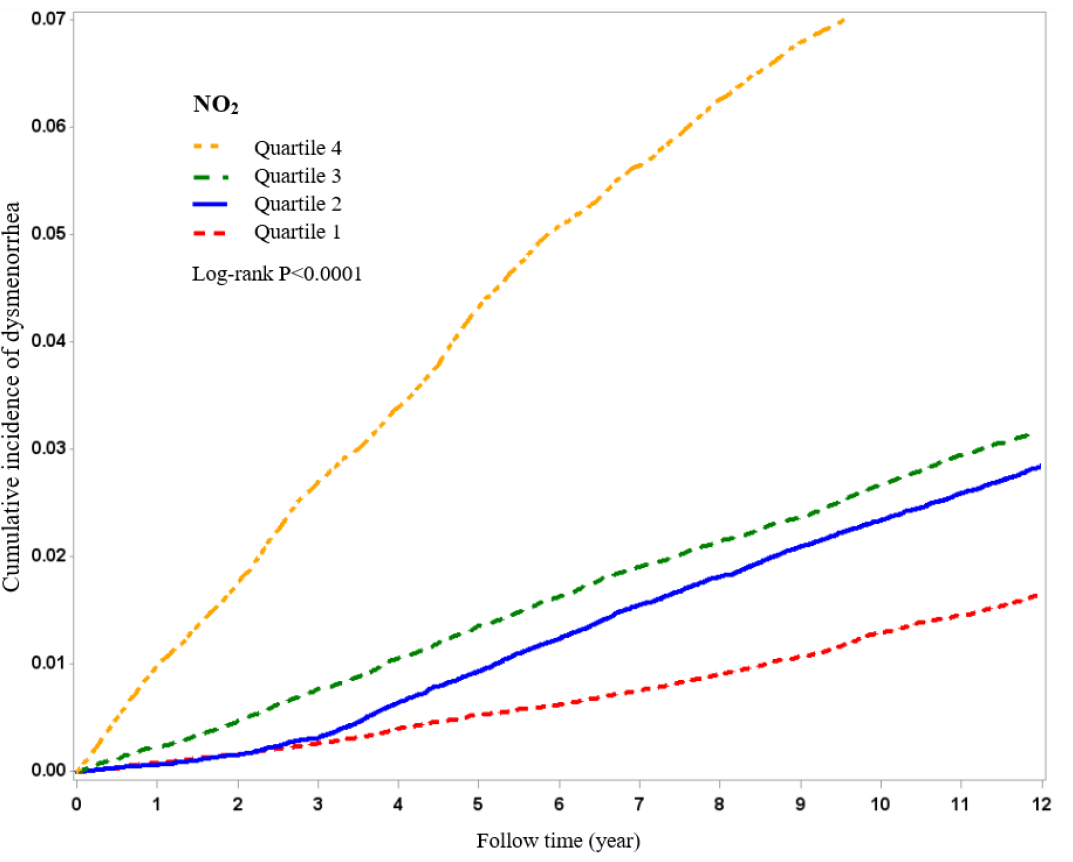

Supplement: Supplementary Figure 5 — Kaplan-Meier curve about NO2 exposure and dysmenorrhea risk. [file Image_5.TIF]

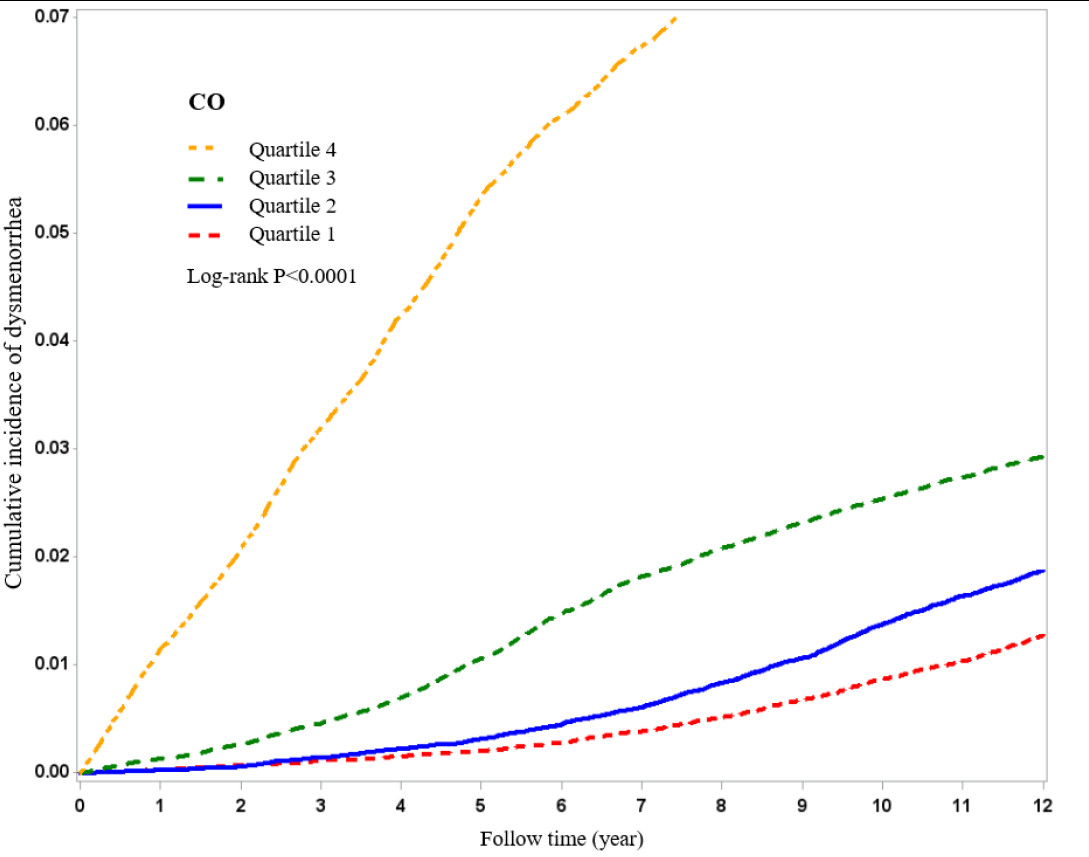

Supplement: Supplementary Figure 6 — Kaplan-Meier curve about CO exposure and dysmenorrhea risk. [file Image_6.TIF]

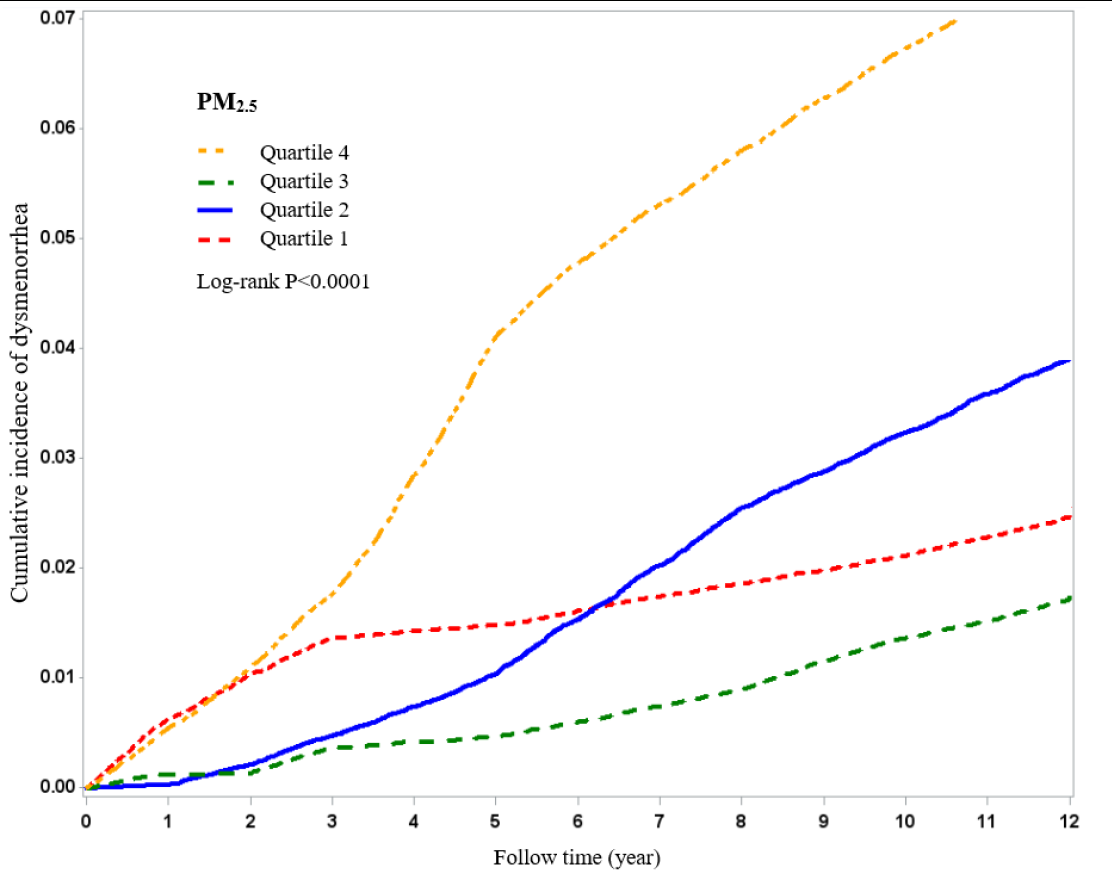

Supplement: Supplementary Figure 7 — Kaplan-Meier curve about PM2.5 exposure and dysmenorrhea risk. [file Image_7.TIF]
