# Supplementary material for: Increased Incidence of Dysmenorrhea in Women Exposed to Higher Concentrations of NO, NO2, NOx, CO, and PM2.5: A Nationwide Population-Based Study
Source: Front Public Health. 2021 Jun 17;9:682341. doi: 10.3389/fpubh.2021.682341 (PMC8247898; doi:10.3389/fpubh.2021.682341)
Supplement: Supplementary Table 1 — Description correlation matrix for air pollutants. ***correlation is significant at the <0.0001 level (two tails). [file Table_1.docx]

Supplementary table 1.Description correlation matrix for air pollutants.

|  | **NOx** | **NO** | **NO2** | **PM2.5** | **CO** |
| --- | --- | --- | --- | --- | --- |
| **NOx** | 1 |  |  |  |  |
| **NO** | 0.966*** | 1 |  |  |  |
| **NO2** | 0.919*** | 0.794*** | 1 |  |  |
| **PM2.5** | -0.131*** | -0.216*** | 0.009*** | 1 |  |
| **CO** | 0.796*** | 0.778*** | 0.723*** | -0.125*** | 1 |

Notes: ***correlation is significant at the <0.0001 level (two tails).
