# Supplementary material for: Increased Incidence of Dysmenorrhea in Women Exposed to Higher Concentrations of NO, NO2, NOx, CO, and PM2.5: A Nationwide Population-Based Study
Source: Front Public Health. 2021 Jun 17;9:682341. doi: 10.3389/fpubh.2021.682341 (PMC8247898; doi:10.3389/fpubh.2021.682341)
Supplement: Supplementary Table 2 — Baseline urbanization level among quartiles of daily average concentration of air pollutants in Taiwan. P-value using chi-square for the comparisons between urbanization level among quartiles of daily average concentration of air pollutants. The urbanization level was categorized by the population density of the residential area into 4 levels, with level 1 as the most urbanized and level 4 as the last urbanized. The daily average air pollutant concentrations were categorized based on quartiles for each air pollutants. [file Table_2.docx]

Supplementary table 2. Baseline urbanization level among quartiles of daily average concentration of air pollutants in Taiwan.

| **Air pollutant concentration** | **Quartile 1 (Q1) (lowest)** | | **Quartile 2 (Q2)** | | **Quartile 3 (Q3)** | | **Quartile 4 (Q4) (highest)** | | **p-value** |
| --- | --- | --- | --- | --- | --- | --- | --- | --- | --- |
| **N=296,078** | **n** | **%** | **n** | **%** | **n** | **%** | **n** | **%** |  |
| **NOx** |  |  |  |  |  |  |  |  |  |
| **Urbanization level** |  |  |  |  |  |  |  |  | <0.0001 |
| 1 (highest) | 8782 | 11.2 | 3021 | 4.21 | 29964 | 41 | 57140 | 78.7 |  |
| 2 | 21516 | 27.4 | 32618 | 45.4 | 25531 | 34.9 | 10861 | 14.9 |  |
| 3 | 13985 | 17.8 | 17862 | 24.9 | 15308 | 20.9 | 4511 | 6.21 |  |
| 4 (lowest) | 34265 | 43.6 | 18329 | 25.5 | 2308 | 3.16 | 77 | 0.11 |  |
| **NO** |  |  |  |  |  |  |  |  |  |
| **Urbanization level** |  |  |  |  |  |  |  |  | <0.0001 |
| 1 (highest) | 8855 | 11.2 | 5578 | 7.75 | 27615 | 43 | 56859 | 70.4 |  |
| 2 | 19630 | 24.8 | 35101 | 48.8 | 17476 | 27.2 | 18319 | 22.7 |  |
| 3 | 14252 | 18 | 16530 | 23 | 15550 | 24.2 | 5334 | 6.61 |  |
| 4 (lowest) | 36511 | 46.1 | 14727 | 20.5 | 3546 | 5.52 | 195 | 0.24 |  |
| **NO_2_** |  |  |  |  |  |  |  |  |  |
| **Urbanization level** |  |  |  |  |  |  |  |  | <0.0001 |
| 1 (highest) | 6913 | 8.8 | 5891 | 8.65 | 25014 | 32.5 | 61089 | 84.5 |  |
| 2 | 22403 | 28.5 | 23775 | 34.9 | 38566 | 50 | 5782 | 7.99 |  |
| 3 | 11990 | 15.3 | 22029 | 32.3 | 12308 | 15.9 | 5339 | 7.38 |  |
| 4 (lowest) | 37262 | 47.4 | 16401 | 24.1 | 1201 | 1.56 | 115 | 0.16 |  |
| **PM2.5** |  |  |  |  |  |  |  |  |  |
| **Urbanization level** |  |  |  |  |  |  |  |  | <0.0001 |
| 1 (highest) | 24358 | 30.8 | 33217 | 47.3 | 27606 | 37.1 | 13726 | 18.9 |  |
| 2 | 28685 | 36.3 | 15478 | 22.1 | 19535 | 26.2 | 26828 | 37 |  |
| 3 | 16557 | 20.9 | 11993 | 17.1 | 9502 | 12.8 | 13614 | 18.8 |  |
| 4 (lowest) | 9378 | 11.9 | 9519 | 13.6 | 17768 | 23.9 | 18314 | 25.3 |  |
| **CO** |  |  |  |  |  |  |  |  |  |
| **Urbanization level** |  |  |  |  |  |  |  |  | <0.0001 |
| 1 (highest) | 6909 | 9.18 | 8165 | 11.8 | 41212 | 54.4 | 42621 | 56.2 |  |
| 2 | 20909 | 27.8 | 25774 | 37.2 | 20740 | 27.4 | 23103 | 30.4 |  |
| 3 | 13081 | 17.4 | 17634 | 25.5 | 11451 | 15.1 | 9500 | 12.5 |  |
| 4 (lowest) | 34324 | 45.6 | 17702 | 25.6 | 2304 | 3.04 | 649 | 0.86 |  |

P-value using chi-square for the comparisons between urbanization level among quartiles of daily average concentration of air pollutants.

The urbanization level was categorized by the population density of the residential area into 4 levels, with level 1 as the most urbanized and level 4 as the last urbanized.

The daily average air pollutant concentrations were categorized based on quartiles for each air pollutants.
